# Direct effects of organic pollutants on the growth and gene expression of the Baltic Sea model bacterium *Rheinheimera* sp. BAL341

**DOI:** 10.1111/1751-7915.13441

**Published:** 2019-07-04

**Authors:** Christofer M. G. Karlsson, Elena Cerro‐Gálvez, Daniel Lundin, Camilla Karlsson, Maria Vila‐Costa, Jarone Pinhassi

**Affiliations:** ^1^ Centre for Ecology and Evolution in Microbial Model Systems EEMiS Linnaeus University Stuvaregatan 4 Kalmar 39231 Sweden; ^2^ Department of Environmental Chemistry IDAEA‐CSIC Jordi Girona 18‐26 Barcelona 08034 Catalunya Spain

## Abstract

Organic pollutants (OPs) are critically toxic, bioaccumulative and globally widespread. Moreover, several OPs negatively influence aquatic wildlife. Although bacteria are major drivers of the ocean carbon cycle and the turnover of vital elements, there is limited knowledge of OP effects on heterotrophic bacterioplankton. We therefore investigated growth and gene expression responses of the Baltic Sea model bacterium *Rheinheimera* sp. BAL341 to environmentally relevant concentrations of distinct classes of OPs in 2‐h incubation experiments. During exponential growth, exposure to a mix of polycyclic aromatic hydrocarbons, alkanes and organophosphate esters (denoted MIX) resulted in a significant decrease (between 9% and 18%) in bacterial abundance and production compared with controls. In contrast, combined exposure to perfluorooctanesulfonic acids and perfluorooctanoic acids (denoted PFAS) had no significant effect on growth. Nevertheless, MIX and PFAS exposures both induced significant shifts in gene expression profiles compared with controls in exponential growth. This involved several functional metabolism categories (e.g. stress response and fatty acids metabolism), some of which were pollutant‐specific (e.g. phosphate acquisition and alkane‐1 monooxygenase genes). In stationary phase, only two genes in the MIX treatment were significantly differentially expressed. The substantial direct influence of OPs on metabolism during bacterial growth suggests that widespread OPs could severely alter biogeochemical processes governed by bacterioplankton.

## Introduction

Organic pollutants (OPs) include both persistent organic pollutants (POPs) and semivolatile organic compounds (SOCs), several of which are partially resistant to degradation in the environment. The awareness of the environmental threat posed by OPs on human health and wildlife emerged after massive usage during World War II (El‐Shahawi *et al*., 2010), but it was not until the Stockholm convention in 2004 that the first global treaty for actions against OPs was signed (UNEP 2001). Initially, 12 POPs, ‘the dirty dozen’, were selected for elimination and restriction worldwide, including the dichlorodiphenyltrichloroethane (DDT) and polychlorinated biphenyls (PCB). Since then, new substances have been continuously added to the list (UNEP 2010, 2017) and there are several thousands of emerging chemicals left to be considered (Muir and Howard, 2006; Ashraf, 2017). A contributing factor to the wide distribution of OPs is that many of them can be transported long distances in the atmosphere and hence become deposited in ecosystems far from their source. For example, atmospheric deposition supports the baseline OP concentrations found in the oceans (Kannan *et al*., 2001; Yamashita *et al*., 2005; Lohmann *et al*., 2007; Castro‐Jimenez *et al*., 2014; González‐Gaya *et al*., 2016). Hydrocarbons, such as alkanes and polycyclic aromatic hydrocarbons (PAHs), have been well studied in the environment and enter marine ecosystems in a number of ways, including atmospheric deposition following burning of fossil fuels and oil spill events (Duran and Cravo‐Laureau, 2016; González‐Gaya *et al*., 2016; Joye *et al*., 2016). Organophosphates are increasingly used as a substitutes for the now‐banned brominated based flame retardants (van der Veen and de Boer, 2012) and as plasticizers (OPEs‐FL‐P). In the marine environment, organophosphates are ubiquitously found at nano‐ to microgram concentrations (Hu *et al*., 2014; Li *et al*., 2017; McDonough *et al*., 2018; Vila‐Costa *et al*., 2019) that are orders of magnitude larger than what has been estimated based on atmospheric inputs (Castro‐Jimenez *et al*., 2014, 2016). Also, perfluorinated compounds (including perfluoroalkyl acids [PFFA]), which are used for example in fire‐fighting foam and textile industry, are of great environmental concern (Lau *et al*., 2007; Wei *et al*., 2015). The latter compounds are sometimes referred to as ‘swimmer OPs’ since an important route for them to reach the oceans is through riverine run‐off (but also potentially through sea‐salt aerosols) (Casal *et al*., 2017b). It is widely recognized that toxic OPs accumulate in food chains due to their hydrophobicity, and the effects of specific pollutants on higher marine life are extensively studied (Hylland, 2006; Schwarzenbach *et al*., 2010; Keyte *et al*., 2013; Harmon, 2015). However, knowledge of the effects on microorganisms of the myriad of OPs (including POPs and SOCs) circulating in the marine environment remains scarce (Dachs and Méjanelle, 2010; Cerro‐Galvez *et al*., 2019).

Bacterioplankton are a key component of marine ecosystems, where they are responsible for the transformation of organic and inorganic nutrients in the water column (Azam, 1998; Falkowski *et al*., 2008). The bacteria play a central role in the biogeochemical carbon cycle since they are the main consumers of dissolved organic carbon (DOC), one of the largest reservoirs of carbon in seawater (Azam *et al*., 1994), and they are also expected to play a major role in the cycling of OPs (Del Vento and Dachs, 2002; González‐Gaya *et al*., 2019). Hydrocarbons in the marine environment can stimulate the growth rates of bacteria and also select for specific bacterial groups (for example members of the genera *Cycloclasticus*,* Marinobacter*,* Oceanospirillum*) (Zobell, 1950; Leahy and Colwell, 1990; Teira *et al*., 2007; Seo *et al*., 2009; Rivers *et al*., 2013). Broadly considered, OPs can influence microorganisms by stimulating growth (acting as a food source) or by negatively affecting growth as a result of toxicological effects (Singh and Walker, 2006; Echeveste *et al*., 2010a, 2010b; Duran and Cravo‐Laureau, 2016; Fernandez‐Pinos *et al*., 2017; Cerro‐Galvez *et al*., 2019; Vila‐Costa *et al*., 2019). Recent research has used advanced molecular methods to identify the mechanisms used by natural bacterial communities to degrade toxic linear or branched hydrocarbons (alkanes) and PAH from oil spill events (Rivers *et al*., 2013; Wang *et al*., 2016; Handley *et al*., 2017). Still few studies have dealt with the direct effects of OPs on bacterial growth, production or metabolic functions under controlled experimental conditions and in response to background concentrations of OPs typically found in surface seawater.

The aim of this study was to explore responses in central and specific metabolic pathways of a model Baltic Sea bacterium when exposed to distinct OP families. We selected *Rheinheimera* sp. strain BAL341 (*Gammaproteobacteria*) as experimental organism because it represents a taxon that is highly responsive to changes in environmental conditions, both in the Baltic Sea and elsewhere (Pinhassi and Berman, 2003; Lindh *et al*., 2015a, 2015b). The OP families represented here were PAHs, alkanes (hydrocarbons), organophosphates (OPEs) and perfluoroalkylated substances (PFASs), all of which are widespread in the marine environment. We hypothesized that experimental analysis of the interaction between different OPs with the bacterial isolate would allow discriminating both positive and negative direct effects of the OPs on growth and metabolism.

## Results

### Brief description of the *Rheinheimera* sp. BAL 341 genome

The *Rheinheimera* sp. strain BAL341 is a Gamm*aproteobacteria* from the Baltic Sea with a fairly large genome size of 4.1 Gbase pairs. The genome of BAL341 contains 3737 putative open reading frames (ORFs), of which 1611 (43%) got a SEED annotation, with an additional 1134 ORFs (30%) getting a functional annotation but not represented in SEED. The remaining 992 ORFs (27% of the protein‐coding genes) were annotated as hypothetical proteins. Interestingly, the bacterium possesses the gene encoding alkane‐1 monooxygenase involved in degradation of alkanes, but lacks dioxygenases genes which are key enzymes for degradation of PAHs.

### Bacterial growth in batch cultures used for pollutant experiments

**Figure 1:**
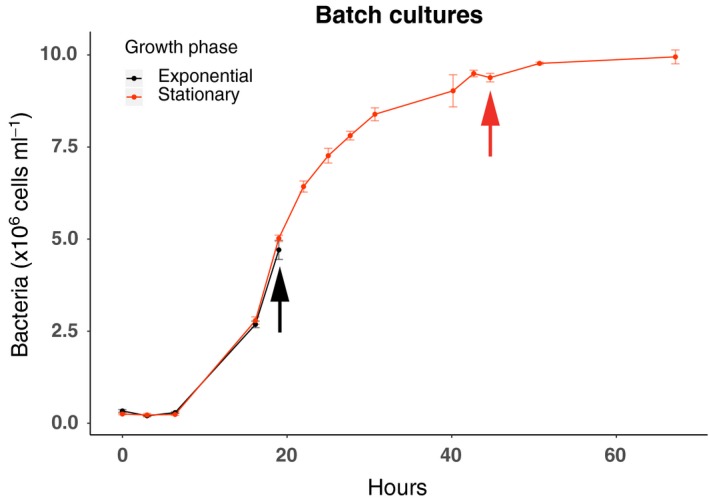
The average bacterial abundance in cells ml^−1^ in the eight batch cultures. Black arrow shows when bacteria were transferred and exposed to OPs in exponential growth phase (~19 h). Red arrow shows when bacteria were transferred and exposed to OPs in early stationary growth phase (~ 44 h). Error bars denote standard deviations of triplicate biological replicates.

Batch cultures used for the pollutant exposure experiments were initiated with bacterial cell numbers averaging 2.9 × 10^5^ (± 0.5 × 10^5^) cells ml^−1^. Bacterial numbers increased to 1.0 × 10^7^ (± 0.03 × 10^7^) cells ml^−1^, following a classical sigmoid growth pattern with a short lag phase of ~6 h and entering stationary growth phase after ~40 h (Fig. 1). From these cultures, subsamples were retrieved for OP exposure experiments in exponential phase (19 h) and in early stationary phase (44 h).

### Growth responses to OPs in exponential phase

Bacterial abundance at *T* = 0 of the exponential phase OP exposure experiment was 4.7 × 10^6^ (± 0.1 × 10^6^) cells ml^−1^. During the 2‐h experiment, bacterial abundance in the controls increased 27% (reaching 6.0 × 10^6^ cells ml^−1^) (Table 1). Linear mixed‐effects model analysis for repeated measures data (LMM) showed that OP exposure had a significant effect on the bacterial abundance (LMM, effect of cell abundance: *F*_2,6_ = 5.2, *P* = 0.049). This overall effect resulted from bacterial abundance in the MIX treatment being significantly lower compared with controls (LMM; effect of cell abundance: *T* [6] = −2.56, *P* = 0.042; Table 1). Bacterial abundance in the PFAS treatment was similar to the controls (LMM, effect of cell abundance: *T* [6] = 0.41, *P* = 0.696; Table 1). Moreover, a post hoc test (Tukey) showed that bacterial abundance in the MIX treatment was also significantly lower than in the PFAS treatment (*P* = 0.008). OP exposure also had a significant effect on bacterial production (LMM; effect of bacterial production: *F*_2,6_ = 11.44, *P* < 0.009), as a result of the bacterial production in the MIX treatment being significantly lower than in the controls (LMM; effect of bacterial production: *T* [6] = −2.88, *P* = 0.028; Table 1). Furthermore, post hoc (Tukey) test showed that the bacterial production in the MIX treatment also was significantly lower than in the PFAS treatment (*P* = < 0.001).

**Table 1:** Bacterial abundance and bacterial production after exposure to the OP treatments MIX and PFAS compared with controls in *T* = 0 h, *T* = 1 h and *T* = 2 h.

	Bacterial abundance (cells ml^−1^)			Bacterial production (pmol Leu l^−1 ^h^−1^)	
	*T* = 0	*T* = 1	*T* = 2	*T* = 1	*T* = 2
	Exponential growth phase				
Control	4.7 × 10^6^ ± 0.1 × 10^6^	5.8 × 10^6^ ± 0.1 × 10^6^	6.0 × 10^6^ ± 0.3 × 10^6^	155 ± 6	166 ± 11
MIX*, a		5.3 × 10^6^ ± 0.2 × 10^6^	5.8 × 10^6^ ± 0.4 × 10^6^	133 ± 9	144 ± 16
PFAS		5.8 × 10^6^ ± 0.2 × 10^6^	6.2 × 10^6^ ± 0.2 × 10^6^	170 ± 21	178 ± 11
	Stationary growth phase				
Control	9.4 × 10^6^ ± 0.4 × 10^6^	10.0 × 10^6^ ± 0.2 × 10^6^	9.9 × 10^6^ ± 0.2 × 10^6^	2203 ± 59	2571 ± 84
MIX		9.7 × 10^6^ ± 0.2 × 10^6^	9.7 × 10^6^ ± 0.4 × 10^6^	1785 ± 159	2118 ± 147
PFAS		9.8 × 10^6^ ± 0.1 × 10^6^	10.0 × 10^6^ ± 0.1 × 10^6^	2103 ± 233	2503 ± 217

^a^Differed significantly from both controls and the PFAS treatment in bacterial abundance and production.

*Significant *P*‐values (*P *<* *0.05) using linear mixed‐effects model for repeated measures.

### Growth responses to OPs in early stationary phase

In the stationary phase OP exposure experiments, bacterial abundance increased from 9.4 × 10^6^ (± 0.4 × 10^6^) cells ml^−1^ at *T* = 0 to 1.0 × 10^7^ (± 0.02 × 10^7^) at *T* = 1, after which abundance remained stable. No statistical differences in bacterial abundance (LMM; effect of bacterial abundance: *F*_2,6_ = 1.73 *P* = 0.254) or production (LMM; effect of bacterial production: *F*_2,6_ = 2.68, *P* = 0.147) were found between the treatments and controls.

### Overview of gene expression patterns in exponential and stationary phase experiments

Transcript sequencing from the exponential and stationary phase experiments on average resulted in 22.9 million sequence reads per sample (range 17.6–43.9 million). After mapping the reads to the genome, we obtained an average of 2.6 million reads annotated to protein‐coding genes per sample in the SEED database (Table 2).

**Table 2:** The number of reads from sequencing and SEED annotation in the different treatments and growth phases (ranges in million reads for triplicate samples from each sample type, except controls from exponential phase which is in duplicates).

	Million raw reads from sequencing		Million reads annotated (SEED)	
	Exponential phase	Stationary phase	Exponential phase	Stationary phase
Controls	17.59–17.73	18.38–23.17	2.02–3.45	1.33–1.87
MIX	21.85–43.89	19.79–25.42	1.85–5.13	1.5–3.56
PFAS	19.43–23.71	20.98–25.98	3.1–4.7	1.12–1.46
Average	22.9		2.6	

**Figure 2:**
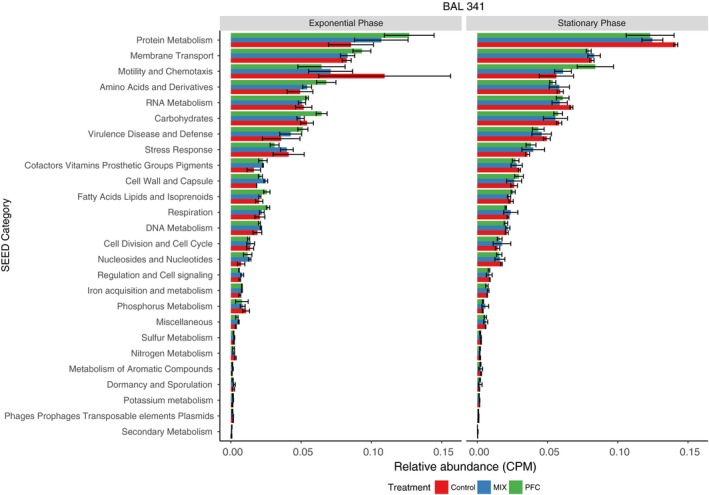
Overview of dominant functions according to the highest subsystem in the SEED classification (*Y*‐axis), in exponential and stationary growth phase, for BAL341 when exposed to MIX or PFAS compared with controls. *X*‐axis shows the relative expression values in counts per million (CPM).

At the top level of the SEED classification hierarchy category, ‘Protein metabolism’ was the dominant category in both the exponential (8.5–12.0%) and stationary phase (12.0–14.0%) (Fig. 2). ‘Membrane transport’ was the second most abundant category with about 8.0% in both growth phases. It can be noted that the category ‘Motility and Chemotaxis’ made up 10.9% of total transcripts in the exponential phase but only 5.6% in stationary phase (Fig. 2). Other major categories included ‘Amino Acids and Derivatives’, ‘RNA metabolism’, ‘Carbohydrates’ and ‘Stress Response’, each of which represented 3.0–8.0% of transcripts.

Inspection of the top 100 individual most abundant genes in the different treatments and growth phases showed that the genes encoding ‘TonB dependent receptor’ and ‘Alkaline serine protease’ were among the top five most expressed genes in all samples. In fact, 45% of the top 100 genes were the same irrespective of treatments and growth phases (Table S2). A majority of the abundant genes were associated to transcription, translation and ribosomal proteins, including for example ‘DNA‐directed RNA polymerase beta subunit (EC 2.7.7.6)’ and ‘Translation elongation factor G/Tu’. Among the abundant genes, ‘Flagellin protein FlaA/FlaG’, ‘Flagellar hook‐associated protein FLiD’ and ‘Cold shock protein CspA/CspD’ can be noted.

To verify if expected rearrangements in expression took place between the exponential and stationary phase cells (Fig. 1), we carried out a statistical analysis of differential gene expression in the control cultures using EdgeR. This showed that, although the top 100 genes were mostly similar among treatments and controls and over time, 1200 genes were significantly different between the two growth phases. Thus, there were substantial differences in expression between the growth phases, consistent with the known major adaptations in physiology and expression to changes in growth conditions among bacteria with moderate‐ to large‐sized genomes (Navarro Llorens *et al*., 2010).

### Comparison of number of differentially expressed genes in the exponential and stationary phase experiments

To determine how many genes that responded to the OP exposures, we statistically examined the differential gene expression using EdgeR. This analysis identified a total of 809 genes that differed significantly between the treatments in the exponential growth phase (Table 3). In the PFAS treatment, 458 genes were significantly differentially expressed compared with controls. Among these genes, 247 genes had a relative abundance higher than in the controls (hereafter referred to as ‘up’) and 211 genes had a relative abundance lower than in the controls (hereafter referred to as ‘down’). In the MIX, 325 genes were significantly differentially expressed compared with the controls (135 up and 190 down). Comparison of gene expression levels between the two OP treatments showed 26 genes that significantly differed between PFAS and MIX (three genes expressed at higher relative abundance in PFAS and 23 genes higher in MIX). In notable contrast, in stationary phase, only two distinct genes were significantly differentially expressed between MIX and controls or between pollutants (Table 3); these genes were higher in MIX.

**Table 3:** Number of differentially expressed genes in the different treatments and growth phases

	Control	MIX	PFAS	
Control		2	0	Stationary phase (total 4)
MIX	325		2	
PFAS	458	26		
	Exponential phase (total 809)			

### Overall analysis of differentially expressed genes in exponential phase

**Figure 3:**
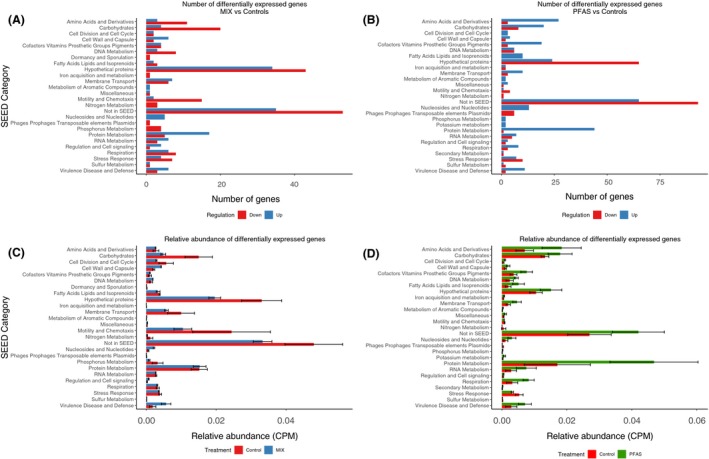
Overview of significantly differentially expressed genes in their respective SEED categories (*Y*‐axis) in exponential growth phase. A. The number of genes expressed at significantly higher (‘up’) or lower (‘down’) relative abundance levels in the MIX treatment compared with controls. B. The number of genes expressed at significantly higher (‘up’) or lower (‘down’) relative abundance levels in the PFAS treatment compared with controls. C. Relative expression levels (CPM) in the MIX treatment and controls. D. Relative expression levels (CPM) in the PFAS treatment and controls.

We further investigated the significantly differentially expressed genes by examine their distribution among SEED categories (Fig. 3). For most SEED categories, the distribution of genes that were found to be expressed at significantly higher (‘up’) or lower (‘down’) relative abundance levels compared with the controls in the PFAS and MIX treatments were fairly similar. Nevertheless, pronounced differences were found for example in the category of ‘Amino acids and Derivatives’, where 11 and 3 genes were down and up respectively, in the MIX (Fig. 3A), whereas 3 and 27 genes were down and up, respectively, in PFAS (Fig. 3B). Similar differences were found in the category ‘Carbohydrates’ (Fig. 3A) where the majority of genes (20) were down and only four were up in the MIX, whereas eight genes were down and 20 genes were up in PFAS. Pronounced differences were also found in the ‘Motility and Chemotaxis’ and ‘Fatty Acids Lipids and Isoprenoids’ categories (Fig. 3A and B). Strikingly, the four significant ‘Phosphorus metabolism’ genes were all down in the MIX (which contains organophosphate esters) compared with controls (Fig. 3A); in PFAS, the two significant genes in this category were both up (Fig. 3B).

There were also important differences in the relative expression levels (i.e. counts per million (CPM)) of the significantly differentially expressed genes (Fig. 3C and D). For example, summed expression levels of significantly differentially expressed genes in the category ‘Motility and Chemotaxis’ were lower in the MIX (1.0 × 10^−2^ CPM) compared with the controls (2.4 × 10^−2^ CPM) (Fig. 3C). The few significant genes in PFAS in this category only reached about 0.07 × 10^−2^ CPM (Fig. 3D). Similar patterns with lower expression levels in the MIX compared with controls were found in the categories ‘Phosphorus metabolism’ and ‘Cell division and cell cycle’ (Fig. 3C). In contrast, higher summed expression levels in the PFAS treatment compared with controls were recorded for the significant genes in the top‐level SEED groups ‘Amino acids and derivatives’, ‘Carbohydrates’ and ‘Cofactors vitamins prosthetic groups pigments’ (Fig. 3D).

### Comparison of shared and unique genes for the OP treatments in exponential phase

**Figure 4:**
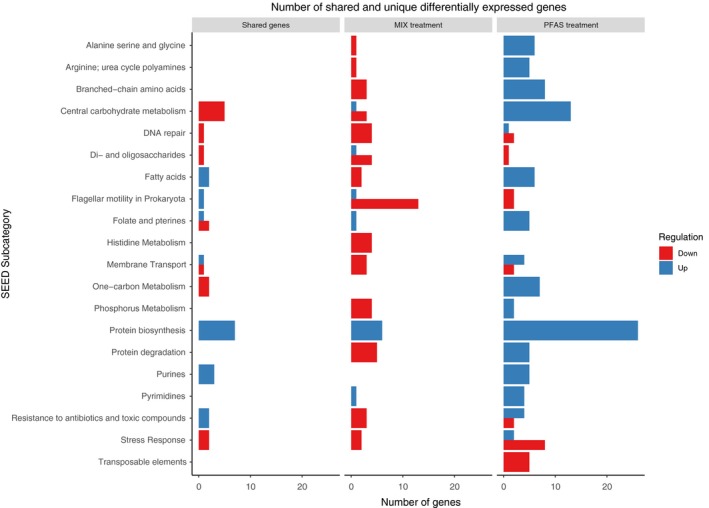
The number of shared and unique significantly differentially expressed genes for the pollutants in their respective SEED subcategory (*Y*‐axis). The red and blue colours represent genes expressed at significantly higher (‘up’) or lower (‘down’) relative abundance levels compared with controls. Only SEED subcategories containing three or more genes are shown.

To further investigate the bacterial response to the different pollutants, we analysed which of the significantly differentially expressed genes that were shared and unique for the pollutants, if they were expressed at higher or lower relative expression levels compared with the controls, and which second level SEED categories they belonged to (Fig. 4). In total, 103 significantly differentially abundant genes were shared between the OP treatments, whereas 222 and 355 were unique for MIX and PFAS respectively. Shared genes were distributed over a broad diversity of SEED categories (46 categories, in total, with 11 categories containing at least three genes). Among the shared genes, similar numbers of genes were significantly up or down compared to controls. In contrast, most of the genes that were significantly expressed uniquely in the MIX were down compared to controls, whereas most genes that were significant uniquely in the PFAS treatment were up compared to controls (Fig. 4). For the unique genes, notable differences between the pollutants could be found in for example the ‘Central carbohydrate metabolism’, ‘Fatty acids’ and ‘Flagellar motility in Prokaryotes’ categories (Fig. 4).

To identify the genes that were most responsive in the MIX and PFAS treatments compared with the controls, we sorted the 809 significantly differentially expressed genes according to their combined logCPM and logFC values and inspected the 50 genes ranked highest and lowest in each treatment (Tables S3 and S4 list all significant genes in the MIX and PFAS treatments compared with the controls respectively). In both treatments, the majority of the top 50 genes were associated with general growth regulation, including tRNA synthesis, protein synthesis, ribosomal protein and the TCA cycle. Nevertheless, in the top of this list in the MIX treatment, we found genes encoding the ‘Cold shock protein CspA’ (also among the top 50 in PFAS), ‘Cell division trigger factor’ and ‘alkane‐1 monooxygenase’ (Table S3), which are key proteins involved in general stress response, cell division during growth, and alkane degradation respectively. Among the 50 genes with strongest decrease in the MIX compared with the control, we found ten genes involved in flagellar motility and three involved in phosphate transport (Table S3).

In the PFAS treatment (Table S4), genes of particular interest were those encoding ‘TonB dependent receptor’ involved in uptake of polymeric substrates, ‘Outer membrane vitamin B12 receptor BtuB’ which mediates high affinity binding and transport of vitamin B12 in a manner dependent on TonB receptors, ‘Peptide transport system permease protein SapC’ typically induced to promote resistance to antimicrobial peptides, and ‘Cold shock protein CspA’ (as in the MIX). Curiously, genes encoding Tn7 transposition proteins TnsBCD and a gene for glutamate synthase (involved in amino acid synthesis and nitrogen metabolism) were expressed at much lower levels in both MIX and PFAS compared with the controls (Tables S3 and S4).

### Overall analysis of differentially expressed genes in stationary phase

In stationary phase, only two genes, encoding ‘Sensory subunit of low CO_2_‐induced protein complex’ and ‘alkane‐1 monooxygenase (EC 1.14.15.3)’ (not placed in SEED categories), were significantly differentially expressed. These two genes were upregulated in the MIX compared with both the PFAS treatment and the controls (Table 3). Interestingly, as noted above, the alkane‐1 monooxygenase gene was also up in the MIX in the exponential phase experiment.

## Discussion

A multitude of OPs circulate in marine environments, and the toxicity of at least some classes of OPs for multicellular marine organisms, in particular animals, has been explored (e.g. Harmon, 2015 and references therein). However, knowledge about the potential effects of OPs at oceanic background concentrations on heterotrophic bacteria is strongly limited (González‐Gaya *et al*., 2019). Our findings demonstrate substantial effects of OPs on bacterial growth, production and gene expression of the Baltic Sea heterotrophic marine model bacterium *Rheinheimera* sp. strain BAL341. In accordance with the general recognition that bacteria are more resistant to environmental stressors during stationary phase (Gorden and Small, 1993; Kolter *et al*., 1993), the OP responses were primarily observed in during active growth. For example, there was a strong increase in relative expression in both MIX and PFAS exponential phase samples of the gene encoding cold shock protein CspA, a protein with a long established and wide role in bacterial stress responses (Keto‐Timonen *et al*., 2016). Generally, the gene expression responses were detected in a variety of categories of gene functions (recorded both as number of genes influenced in different categories and as their relative expression levels), indicating that the studied OPs had a broad impact on bacterial physiology. Moreover, as much as 85% of the significantly differentially expressed genes were unique for each pollutant treatment, suggesting a high degree of pollutant‐specific physiological responses in marine bacteria (Cerro‐Galvez *et al*., 2019).

The OPs used in our MIX treatment are proven to be degradable and hence can be used as a resource for bacteria (Leahy and Colwell, 1990; Takahashi *et al*., 2008, 2010, 2012, 2017; Seo *et al*., 2009; Abe *et al*., 2014, 2017; Joye *et al*., 2016; Kera *et al*., 2016; Vila‐Costa *et al*., 2019)). Yet, our study showed notably negative effects on bacterial growth of hydrocarbons and OPEs supplied at environmentally relevant concentrations. A broad variety of bacteria can grow exclusively or to a large extent on hydrocarbons as a carbon source (currently known for around 300 bacterial genera) (Prince *et al*., 2018). Genes for degradation of PAHs (most notably the important dioxygenases) are broadly distributed among microbes in the global ocean, yet are missing in several genera (Ghosal *et al*., 2016; González‐Gaya *et al*., 2019). The genome of *Rheinheimera* sp. strain BAL341 lacks the canonical PAH degradation dioxygenase genes (and the cytochrome P450‐mediated pathway) and can thus give insights into how bacteria are handling PAHs when lacking the dioxygenase genes. Still, the genome of BAL341 encodes monooxygenase, indicating it could be specialized in alkane degradation (Wang and Shao, 2013). Notably, the relative expression of alkane 1‐monooxygenase in the MIX increased both in the exponential and stationary phase. This enzyme is essential for the initial oxidation step of alkanes, and its increased expression is in line with other studies showing its importance for the degradation route of hydrocarbons in bacterial isolates and natural bacterioplankton assemblages (Rivers *et al*., 2013). Together with the observed decreases in bacterial numbers and production, our findings support the suggestion that microbial cells can be toxically affected even if they have the capacity to break down the compounds (Sikkema *et al*., 1995). It might even be that the degradation itself could act as a defence mechanism (Ramos *et al*., 2002).

Previous research has concluded that increased PAHs and complex mixtures of OPs negatively affect growth of photosynthetic single‐cell cyanobacteria like *Synechococcus* and *Prochlorococcus* (Echeveste *et al*., 2010a, 2010b). The latter organism also showed decreased gene expression for photosynthetic activity (Fernandez‐Pinos *et al*., 2017). Other analyses showed that smaller sized phytoplankton (both cyanobacteria and eukaryotic species) were more vulnerable to PAHs (pyrene and phenanthrene) compared with larger species (e.g. diatoms in the genus *Thalassiosira*) – both in cultured and natural phytoplankton populations exposed to concentrations ranging from 1 to 1000 μg l^−1^ (Echeveste *et al*., 2010a, 2010b). Our findings substantially expand the range of organisms negatively influenced by PAHs in the lower range of these concentrations to include also heterotrophic bacteria.

The microbial responses to combinations of the two OP classes hydrocarbons and OPEs have to our knowledge not been investigated before. As discussed above, this combination could be beneficial to bacteria, since it provides both carbon in the hydrocarbons and phosphorus in the OPEs (Vila‐Costa *et al*., 2019). Indeed, several genes involved in phosphate acquisition decreased in relative expression in the MIX, potentially indicating that phosphorus (P) in OPEs were readily available to BAL341. Since P is an important growth‐limiting nutrient is major sea areas, this suggests that OPEs could act to promote growth of marine bacteria (Vila‐Costa *et al*., 2019). However, it is also plausible that the combination of hydrocarbons and OPEs could have synergistic toxic effects. Recent studies on Cyanobacteria (*Prochlorococcus*) and phytoplankton in general conclude that OP mixtures have synergistic effects and may become more toxic than the single pollutants (Echeveste *et al*., 2016; Fernandez‐Pinos *et al*., 2017). It has also been shown that LC50 values (i.e. the lethal concentration of a chemical where 50% of the total population is diseased) were lower for natural communities than for cultivated isolates (Echeveste *et al*., 2010a, 2010b). If valid also for bacterioplankton, it becomes important to consider that natural bacterial communities may be even more sensitive to OPs than reported in our laboratory experiments.

For the perfluoroalkylated pollutants (PFOS and PFOA), there is very limited information on their influence on bacteria. Bioaccumulation and toxicological effects on zooplankton and phytoplankton have been described (Mhadhbi *et al*., 2012; Casal *et al*., 2017a, 2017b). The chemical bonds between the carbon and fluorine existing in PFOA and PFOS molecules are particularly strong, wherefore it is considered that the potential transformation of such molecules is extremely slow (Smart, 1994). Although there are laboratory studies showing that these compounds can be catalysed by enzymatic systems (Liu and Mejia Avendano, 2013), there are so far no reports on microbial degradation of PFOS and PFOA compounds under natural conditions (Casal *et al*., 2017a, 2017b). Furthermore, a recent attempt to study microbial degradation of PFOS and PFOA (and polyfluorinated homologues) in wastewater sludge showed no conclusive proof for microbial degradation (Ochoa‐Herrera *et al*., 2016). Still, there is research showing that these pollutants cause for example luminescence inhibition in the marine bacterium *Vibrio fischeri* and the cyanobacterium *Anabaena* sp. strain CPB4337 (Rosal *et al*., 2010), cause membrane disruption, oxidative stress and DNA damage in *Escherichia coli* bacteria (Liu *et al*., 2016), and that they influence bacterial membrane fluidity, permeability and quorum sensing (Fitzgerald *et al*., 2018a, 2018b). Furthermore, there is a 16S rRNA gene survey indicating that these compounds can drive selective changes in bacterial sediment communities (Sun *et al*., 2016). In the current study, we found no growth responses of bacteria in exponential phase to the combination of PFOS and PFOA and only a very minor difference for bacteria in stationary phase. This might be because the concentrations of PFASs were too low for giving any lethal/inhibiting effects on growth. Actually, the concentrations used in former experiments where cell death of microorganisms occurred were up to 500 000 times higher (Rosal *et al*., 2010; Liu *et al*., 2016).

Nevertheless, it is noteworthy that, despite no discernable responses in bacterial cell abundance or production, the PFAS treatment induced pronounced differences in gene expression. In fact, the number of genes significantly affected by the PFASs was even higher than that recorded in the MIX treatment where also growth responses were observed – 458 genes compared with 325 genes (Table 3). This shows that there is a strong gene regulation induced due to the exposure to PFAS, and given the key role of gene regulation in determining physiology we infer that upon long term exposure to PFAS pollutants could influence growth. In fact, several studies on gene expression in natural bacterioplankton communities have shown that changes in environmental conditions induce stark changes in gene expression, although detectable changes in growth or other metabolic activities were not detected or only very weak. Such gene expression responses are interpreted as indicative of for example ‘cryptic’ sulfur cycling both in the ocean and in experimental systems with model bacteria (Canfield *et al*., 2010; Durham *et al*., 2015). In an analogous manner, we have previously reported that gene expression analysis is a sensitive measure of pH stress as that predicted to become important under future ocean acidification scenarios caused by continued fossil fuel combustion (Bunse *et al*., 2016). We therefore conclude here that PFASs have a strong influence on bacterial metabolism, and that it will be important to investigate in detail how they may influence bacterial growth and activity in different seas.

## Conclusions and outlook

The use of genomics and transcriptomics is currently becoming an established approach with a sensitive ability to probe metabolic responses of microorganisms in culture and in nature to changes in growth conditions or environmental stressors. Here, we show the usefulness of studying the responses of specific marine model bacteria to organic pollutants and how such model systems can generate testable hypotheses on bacterial responses to different pollutant categories (i.e. thanks to the limited genetic complexity of a particular genome as compared to highly diverse natural communities). Our findings show that genes responding to OPs were involved in several distinct cell functions indicated that these families of pollutants interact with bacterial metabolism through a variety of mechanisms and in a growth‐phase‐dependent manner. More specifically, it appears like the more labile fraction of marine OPs, that is the hydrocarbons and OPEs, rapidly can affect both bacterial growth and gene expression whereas the more degradation‐resistant family, PFAS, induces metabolic cascades that likely require more time before influencing growth. Still, it is important to recognize that different taxa will respond differently to the variety of organic pollutants. Therefore, it is imperative to continue investigating representatives of the myriads of pollutants and how they affect both bacterioplankton communities and key representatives of distinct taxonomic groups of marine microorganisms. Such experiments will be essential to accurately assess the effects of OPs on bacterioplankton regulation of biogeochemical cycles. Experiments like those done here ultimately have the potential to contribute knowledge on genes that could serve as marker/signature genes for OPs circulating in the marine environment.

## Experimental procedures

### Bacterial isolate and culture conditions

*Rheinheimera sp*. strain BAL341 (class *Gammaproteobacteria*) was isolated from surface water (2 m depth) at the Linnaeus Microbial Observatory (LMO), 11 km offshore Kårehamn, in the Baltic Sea (N 56° 55.8540′, E 17° 3.6420′) on 12 of July 2012. BAL341 was spread on Baltic Zobell agar plates (i.e. a mixture of 5 g bacto peptone, 1 g yeast extract and 15 g bacto agar per l of sterile Baltic Seawater, Difco) and transferred to 1 ml Baltic zobell (5 g Bacto peptone and 1 g yeast extract per l of sterile Baltic Seawater, Difco) before being preserved in glycerol (final concentration 25%) and stored at −80°C. DNA from BAL341 for 16S rRNA gene and whole genome sequencing was extracted using the E.Z.N.A. Tissue DNA kit (Omega Bio‐Tek, Norcross, Georgia, USA) following the manufacturer's protocol for extraction of cultured cells in suspension. BAL341 was initially identified through its 16S rRNA gene sequence. The 16S rRNA gene was amplified by using the primers 27F and 1492R (Frank *et al*., 2008) at final concentration of 10 picomole per μl with following PCR thermal cycling program: 95°C for 2 min; 30 cycles of 95°C for 30 s, 50°C for 30 s, and 72°C for 45 s; and 72°C for 7 min. The PCR product was then purified by using E.Z.N.A. Cycle‐Pure Kit (Omega Bio‐Tek, Norcross, Georgia, USA) following the spin protocol according to manufacturer's instruction. Sanger dideoxy sequencing was performed at Macrogen Europe, Amsterdam, Netherlands. The 16S rRNA gene sequence of BAL341 has GenBank accession number KM586890. 

### Preparation of organic pollutants (OPs) mixtures

Two different mixtures of pollutants were used in the experiment; both were obtained by using pure commercial standards. The first mix contained a combination of sodium perfluoro‐1‐[1,2,3,4‐13C4]octanesulfonate acid (MPFOS) and perfluoro‐n‐[1,2,3,4‐13C4]octanoic acid (MPFOA) at final concentration of 1000 and 500 ng l^−1^ respectively. These belong to the functional group of perfluoroalkylated substances (PFASs), thus this combination will hereafter be referred as PFAS. The second mixture, defined as MIX, contained the 16 polycyclic aromatic hydrocarbons (PAHs) regulated by the US Environmental Protection Agency (EPA) spiked at 1000 ng l^−1^, 26 alkanes at 10 000 ng l^−1^ and 9 organophosphate triesters (OPEs) at 1000 ng l^−1^ (see Table S1 for details on compounds). The concentrations of the OPs added to the experimental culture media tentatively reflected the proportion in the marine environment of OPs in relation to the dissolved organic carbon pool available for bacteria (‘tentatively’ since the two parameters are rarely measured in the same sample, but see below). Regarding alkanes and PAHs, one has to consider that the concentrations of the aliphatic and aromatic unresolved complex mixtures (UCMs) are much higher than the concentrations of resolved alkanes and PAHs respectively (Maldonado *et al*., 1999; González‐Gaya *et al*., 2016; Fourati *et al*., 2018). Therefore, the concentrations used for these two families of chemicals are representative of total aliphatic and aromatic hydrocarbons (Cripps, 1992; Boehm *et al*., 2011; Al‐Akhaly *et al*., 2018). In the case of OPEs and PFOS, unfortunately, the total concentrations of P‐ and F‐containing pollutants from the complex mixture are unknown, thus we took the information from field studies quantifying selected compounds from each family. The proportion of the different families of pollutants was 1 PAH : 10 alkanes : 1 OPE : 1 PFOS : 0.5 PFOA, which approximately reflects the occurrence of pollutants usually found in open ocean and coastal areas (Stortini *et al*., 2009; Gonzalez‐Gaya *et al*., 2014; Casal *et al*., 2017a, 2017b; Li *et al*., 2017) although the concentrations can vary across regions (see references for details). The compounds included in the MIX mixture were chosen because previous research has shown that bacterial taxa have the ability to break down these compounds and use them as food source (Singh and Walker, 2006; Joye *et al*., 2016; Vila‐Costa *et al*., 2019). Different enzymes are involved in the degradation of these compounds; therefore, we believe that we could distinguish responses to the compounds genetically.

### Experimental setup, sampling and spiking of pollutants in exponential‐ and stationary growth phase

To investigate the effects of the selected model OP families on the growth and gene expression patterns of Baltic Sea bacteria, we grew the model bacterial isolate *Rheinheimera sp*. strain BAL341 as follows. After taking the isolate from the −80°C freezer, BAL341 were grown on Baltic zobell agar plates for 4 days at room temperature. It was then inoculated into 20 ml Baltic zobell medium in an acid‐washed 100 ml glass bottle and cultivated for 15 h on a shaker (140 rpm; Unimax 2010 orbital shaker, Heidolph) at room temperature (~20°C). Thereafter, 50 μl of overnight culture was transferred to a so‐called ‘intermediate culture’ consisting of a 0.5 l acid‐washed polycarbonate bottle containing 150 ml artificial seawater (seven practical salinity units, prepared from Sea Salts; Sigma) with addition of NH_4_Cl, Na_2_HPO_4_ and Baltic zobell medium for carbon (C) with final concentrations of 0.160 mM N, 0.022 mM P and 1 mM C respectively. After 26 h growth in room temperature, bacteria from the intermediate culture were inoculated into eight replicate batch cultures in pre‐baked (450º, 4 h) glass bottles, each containing 5 l of artificial seawater (seven practical salinity units, prepared from Sea Salts; Sigma) with same nutrient concentrations as the intermediate culture. Bacterial abundance in the eight replicate batch cultures was monitored for 67 h, with sampling roughly every 3 h during daytime (Fig. 1).

Two experiments of pollutant mixture addition (PFAS, MIX and control, see above) were performed at two different stages of the bacterial growth: at the exponential growth phase and at the early stationary phase (Fig. 1). OPs mixtures were added in triplicates pre‐baked empty 2 l glass bottles 2 h before starting each exposure. This was to allow for evaporation and avoid potential toxic effects of the solvent (acetone) used to prepare the mixtures. The same volume of solvent alone was added in triplicates to the control bottles.

For the experiment of OP exposure to exponential growth phase cells, four of the eight replicate batch cultures were transferred and divided equally into the pre‐spiked 9 pre‐baked 2 l glass bottles (where OPs was added as previously described) after 19 h of growth (indicated by black arrow in Fig. 1). For the stationary phase experiment, the remaining four replicate batch cultures were divided similarly after 44 h of growth (indicated by red arrow in Fig. 1). Both the exponential and the stationary phase experiment additions lasted for a total of 3 h and 20 min, of which ~1 h 20 min was time for setting up experiments and the exposure to pollutants thereafter lasting 2 h. Subsamples for bacterial abundance and production were collected after 1 h (denoted *T* = 1) and at 2 h (denoted *T* = 2) of incubation. Samples for gene expression were collected at *T* = 2.

### Bacterial abundance and production

Bacterial abundance samples were collected in triplicates and were fixed with 1% paraformaldehyde (final concentration) and stored frozen at −80°C. Bacterial cells were counted using a flow cytometer Cube 8 (Partec) according to the protocol in Giorgio *et al*. (1996). Bacterial production (BP) was measured using ^3^H‐leucine incorporation rates according to Smith and Azam (1992). For BP, triplicate samples and one killed control were incubated for 1 h in darkness.

### Genome sequencing and annotation

DNA from BAL341 was sequenced with Illumina HiSeq 2500 (PE 2 × 125 bp). Assembly was performed with Spades using default settings (Nurk *et al*., 2013), and annotation was made with the RAST online service (Aziz *et al*., 2008; Overbeek *et al*., 2014) using the SEED system (Overbeek *et al*., 2005). Completeness was estimated using CheckM (Parks *et al*., 2015). The genome is available at EMBL‐EBI with the accession number PRJEB29737.

### RNA sampling, extraction and sequencing

Samples for gene expression analysis (mRNA) were collected in triplicates for the controls and each treatment (i.e. MIX and PFAS) in the exponential and stationary growth phase experiments after 2 h exposure to OPs, by filtering ~2 l of culture on 0.22 μm Sterivex filters (Merck Millipore, Germany) within 20 min. Subsequently, the RNA was stabilized by addition of 2 ml of RNA later (QIAGEN, Sweden) and snap frozen in liquid nitrogen before storage at −80°C until further processing.

Extractions of RNA were completed by thawing the sterivex filters and gently removing RNAlater using a syringe (CODAN,  Rødby, Denmark). Filters were cut open using a clean razor cutter, cut into smaller pieces and transferred to an RNAse‐free microfuge tube (Ambion, Austin, Texas, USA). Thereafter, the extraction continued according to a protocol adapted from Poretsky *et al*. (2009), but using 1.5 g of 200 μm Low binding Zirconium beads (OPS Diagnostics, Lebanon, New Jersey, USA) for bead beating. Briefly, samples were DNase treated using TURBO DNA‐free Kit (ThermoFisher Scientific, Waltham, Massachusetts, USA) and Ribosomal RNA was depleted using RiboMinus Transcriptome Isolation Kit and RiboMinus Concentration Module (ThermoFisher Scientific, Waltham, Massachusetts, USA) according to supplied protocols. RNA was amplified using the MessageAmp II‐Bacteria RNA Amplification Kit (ThermoFisher Scientific, Waltham, Massachusetts, USA) according to manufacturer's instructions. The amplified RNA was quantified with a Qubit 2.0 fluorometer (Invitrogen by Life Technologies, Carlsbad, California, USA), and the RNA quality was checked with a Nanodrop 2000 Spectrophotometer (ThermoFisher Scientific, Waltham, Massachusetts, USA). Sequencing was carried out on the HiSeq 2500 sequencing system (Illumina, San Diego, California, USA) platform [1 × 51 bp] using HiSeq Rapid SBS with the v2 chemistry kit, at Scilifelab, in Stockholm, Sweden. One replicate of the controls in exponential growth phase failed during sequencing and was therefore discarded from further analysis. The RNAseq data is available at EMBL‐EBI with the accession number PRJEB29737.

### Bioinformatical and statistical analysis

Raw RNA sequence reads were quality trimmed with Sickle (https://github.com/najoshi/sickle) and mapped to the assembled BAL341 genome with Bowtie2 (Langmead and Salzberg, 2012). Quantification of ORFs was performed with HTSeq (Anders *et al*., 2015).

To investigate whether bacterial abundance or production was significantly different between treatments after exposure for OPs in exponential and stationary growth phase, a linear mixed‐effects model for repeated measures data was performed (Lindstrom and Bates, 1988) followed by a Tukey post hoc test. All statistical analyses were performed in R (2018), using the EdgeR (Robinson *et al*., 2010), vegan (Oksanen *et al*., 2017), tidyverse (Wickham, 2017), nlme (Pinheiro *et al*., 2018) and ggplot2 (Wickham, 2009) libraries. *P*‐values were set as significant with an *a*‐value of < 0.05.

## Conflict of interest

None declared.

## Author contributions

CMGK and JP conceived the study. CMGK, ECG, MVC and JP designed the study. CMGK and ECG performed the experiment. Laboratory work was done by CMGK and ECG with help from CK. DL and CMGK did the bioinformatics. CMGK and JP wrote the article. All authors read and approved the final manuscript.

## Supporting information


**Table S1.** Detailed list over organic pollutants used in the MIX treatment.

**Table S2.** Gene list showing the most expressed genes in counts per million (CPM) in each treatment and growth phase.

**Table S3.** Gene list showing the top 50 most highly expressed and high fold‐change differentially expressed genes (denoted with positive logFC*logCPM values), and the 50 genes with strongest decrease in relative expression abundance and fold‐change (denoted with negative logFC*logCPM values) in the MIX treatment compared to controls.

**Table S4.** Gene list showing the top 50 most highly expressed and high fold‐change differentially expressed genes (denoted with positive logFC*logCPM values), and the 50 genes with strongest decrease in relative expression abundance and fold‐change (denoted with negative logFC*logCPM values) in the PFAS treatment compared to controls.

## Data Availability

Transcriptomic (rRNA) sequences of *Rheinheimera* sp. BAL341 and the genome as raw and annotated reads are publicly available in the EMBL‐EBI European Nucleotide Archive repository (https://www.ebi.ac.uk/ena), under primary accession PRJEB29737.
